# Correlation between Serum Osteopontin and miR-181a Levels in Allergic Rhinitis Children

**DOI:** 10.1155/2016/9471215

**Published:** 2016-04-21

**Authors:** Wenlong Liu, Qingxiang Zeng, Renzhong Luo

**Affiliations:** Department of Otolaryngology, Guangzhou Women and Children's Medical Center, Guangzhou Medical College, No. 9 Jinsui Road, Guangzhou 510623, China

## Abstract

*Background*. Osteopontin (OPN) has been proved to be associated with allergic airway inflammation. However, the roles of OPN and its regulation in childhood allergic rhinitis (AR) are poorly understood.* Objective*. This study aims to evaluate the expression of OPN and miR-181a in children with AR and their association with Th1/Th2 immune response.* Methods*. Children who suffered from AR were included along with control subjects. Serum was collected to examine the level of OPN and Th1/Th2 cytokines by enzyme-linked immunosorbent assay (ELISA) and the level of miR-181a by quantitative polymerase chain reaction (qPCR).* Results*. Children with AR had significantly higher serum levels of OPN and lower serum levels of miR-181a than healthy controls. Furthermore, serum levels of OPN were positively correlated with Th2 cytokine and negatively correlated with Th1 cytokine. On the contrary, miR-181a level had a negative correlation with IL-4/IL-5 and positive correlation with IFN-*γ*/IL-12. More importantly, there was also significant negative correlation between OPN and miR-181a.* Conclusion*. The OPN protein and miR-181a levels may serve as predictors of disease severity in childhood AR and appear to be promising targets for modulating AR.

## 1. Introduction

Allergic rhinitis (AR) is a common chronic respiratory disease of the upper airway which is characteristically presented with rhinorrhea, sneezing, and congestion [1]. The overall prevalence of AR in children aged 6-7 years and 13-14 years was 8.5% and 14.6%, respectively [2]. Children with AR tend to have poor quality sleep and consequent fatigue and impaired cognitive functioning and academic performance [3]. Moreover, chronic mouth breathing owing to nasal obstruction in AR children is linked to facial abnormalities and dental malocclusions [2]. It has been well accepted that undertreated AR is a major risk factor in asthma development and exacerbation [4]. Enhanced T helper (Th) 2 immune response and eosinophil cells infiltration are considered to be the main pathophysiology features [5].

Osteopontin (OPN) is an extracellular matrix protein and cytokine that could be produced by the airway epithelial cells and inflammatory cells around airways. It is a multifunctional protein in allergy and asthma, an immune modulator that has been recognized as a key regulator in immunoglobulin (Ig) E-mediated and Th2-skewed allergic response [6]. Also, OPN is expressed in human eosinophil followed by GM-CSF and IL-5 activation, which is likely to contribute to the process of angiogenesis in the airways in asthma [7]. Apart from eosinophils, there are other cells that can express OPN in allergic diseases, such as B cells and dendritic cells (DCs). On the other side, OPN also orchestrates DC recruitment to the lung influencing disease [6]. These results suggested that the source and role of OPN are wide and complex. Our previous studies showed that serum OPN level was elevated in patients with AR and might contribute to asthma comorbidity by promoting eosinophil cells migration and activation [8]. An increase in serum OPN levels has also been observed in school-age asthmatic children similar to that seen in asthmatic adults [9]. However, the serum levels of OPN in children with AR were unclear.

MicroRNAs (miRNAs) are small non-protein-coding RNAs that function as posttranscriptional regulators of target genes expression by suppressing translation or through mRNA degradation [10]. Microarray analysis showed that many miRNA expressions in nasal mucosa were altered in AR and differentially expressed miRNAs appear to be involved in the development of AR [11]. Here we hypothesized the involvement of microRNAs in OPN expression at the posttranscriptional level in childhood AR. Of those miRNAs that related to OPN, miR-181a has been indicated to be related to OPN expression in hepatocellular cancer and atherosclerosis [12, 13]. We therefore evaluated the serum levels of OPN and miR-181a in children with AR and investigated their association with the Th1/Th2 cytokines as well as the severity of the disease.

## 2. Methods

### 2.1. Patients

Twenty-five children with AR were recruited at the Department of Otolaryngology, Guangzhou Women and Children's Medical Center, from January 2015 to October 2015. The diagnosis of AR was made on history, clinical examination, skin prick test, and specific IgE measurement according to the Allergic Rhinitis and Its Impact on Asthma (ARIA) guideline (2010) [14]. Twenty children of similar age and gender with no history of allergic diseases were enrolled as the control group. Children were excluded if any of the following criteria were present: history of atopic dermatitis, asthma, nasal abnormalities, and concurrent purulent nasal infection, use of systemic or topical corticosteroids or sodium cromoglycate within the past 4 weeks, use of histamine H1 antagonist within the past 7 days, or a history of any infection within the past 2 weeks. The study protocols were approved by ethics committee boards. Informed consent was obtained from the parents of all subjects. Total nasal symptom score (TNSS) was used in our study to evaluate the symptoms, which is a daily symptom severity score that rates nasal congestion, rhinorrhea, nasal itching, sneezing, and postnasal drip on a 0- to 3-point scale [15].

### 2.2. Blood Sample Collection

Venous blood samples were obtained into Vacuette tubes between 6 am and 8 am after an overnight fast. After centrifuging at 1000 g for 15 min at 4°C, serum samples were separated and stored at −80°C until assay.

### 2.3. Determination of miR-181a Level by Quantitative Real-Time PCR (qRT-PCR)

For miR-181a, total RNA from each serum sample was reverse transcribed into cDNA using the TaqMan microRNA reverse transcription kit and TaqMan microRNA assays (Invitrogen), as per manufacturer's instructions. The results were calculated with the 2^−ΔΔCt^ method and normalized to RNA U6 controls.

### 2.4. Determination of OPN and Th1/Th2 Cytokines by ELISA

The serum levels of OPN, IFN-*γ*, IL-5, IL-12, and IL-4 were measured in duplicate using ELISA kits (R&D, USA) according to the manufacturer's instructions. The detection limits of the assays were as follows: OPN (312.5 pg/mL), IFN-*γ* (12.5 pg/mL), IL-4 (1.56 pg/mL), IL-5 (7.8 pg/mL), and IL-12 (2.5 pg/mL). Eosinophil cationic protein (ECP) was detected by Unicap system.

### 2.5. Statistical Analysis

The data are presented as mean ± SD. The differences between groups were determined by Student's *t*-test. A *P* < 0.05 was set as a significant criterion.

## 3. Results

### 3.1. Demographic and Laboratory Characteristics of the Study Population

We enrolled 25 children with AR and 20 normal controls for the study. Anthropometric parameters of the AR children and controls were listed in Table 1. The mean values for age, weight, height, BMI, and gender distribution did not vary between AR and healthy children.

### 3.2. Increased OPN and Decreased miR-181a in Serum in Children with AR

**Figure 1:**
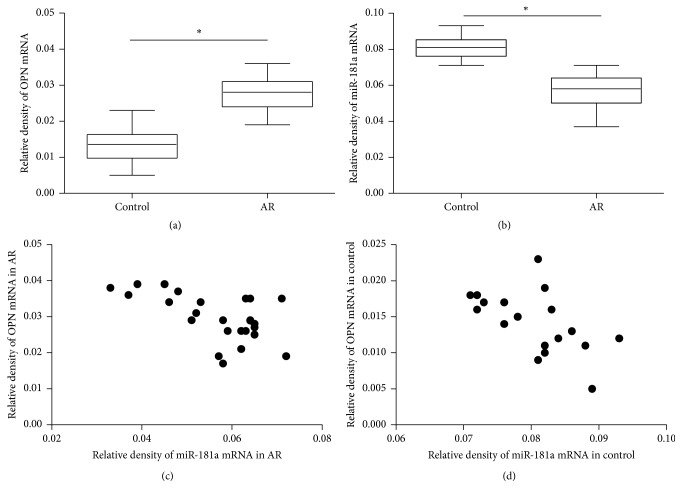
Serum OPN level in the AR group was significantly higher than that in the control group (a). In contrast, serum miR-181a level in the AR group was significantly lower than that in the control group (b). Serum miR-181a level was significantly associated with serum OPN level in both AR (c) and control (d); ^*∗*^ *P* < 0.001.

Concomitant OPN and miR-181a levels in serum were evaluated. As shown in Figure 1, the median serum OPN level in the AR group was significantly higher than that in the control group. In contrast, serum miR-181a level in the AR group was significantly lower than that in the control group. The logistic correlation analysis was used to determine the relationship of OPN and miR-181a in the serum from children with AR. The analysis revealed that serum miR-181a level was significantly associated with serum OPN level in both AR and controls, as shown in Figure 1 (*r* = −0.58, *P* < 0.05; *r* = −0.59, *P* < 0.05).

### 3.3. OPN and miR-181a in relation to Th1/Th2 Cytokines in AR

OPN protein level was positively correlated with Th2 cytokine IL-4/IL-5 and negatively correlated with Th1 cytokine IFN-*γ*/IL-12 (Tables 2 and 3). On the contrary, the serum level of miR-181a had a negative correlation with IL-4/IL-5 and a positive correlation with IFN-*γ*/IL-12 (Tables 2 and 3).

### 3.4. OPN and miR-181a Correlated with Clinical Severity in Children with AR

**Figure 2:**
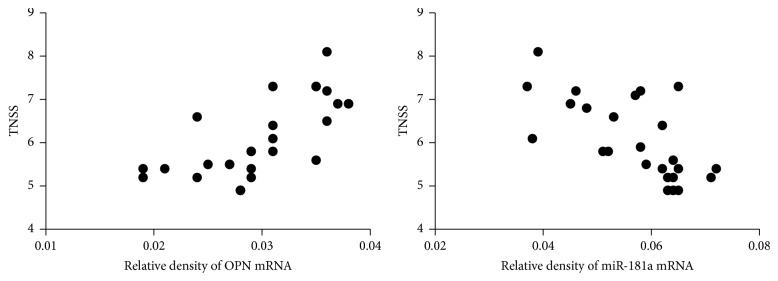
Positive correlation between serum OPN level and total nasal symptom score (TNSS) and negative correlation between serum miR-181a level and TNSS.

To identify if OPN and miR-181a have relationship with the severity of pediatric AR patients, the TNSS of the children was assessed. As shown in Figure 2, there was a significant positive correlation between serum OPN level and TNSS (*r* = 0.68; *P* < 0.05). The results also revealed a significant inverse correlation between serum miR-181a level and TNSS (*r* = −0.66; *P* < 0.05).

## 4. Discussion

There is limited literature regarding the roles of OPN and miRNAs in airway allergic inflammation and most relates to asthma and adult population. In this study, we demonstrated that the serum level of OPN was elevated and miR-181a was decreased in AR children compared to that in normal controls. OPN level was positively correlated with Th2 cytokine IL-4 and total nasal symptom score, whereas miR-181a level had a negative correlation with them. Our key finding was that the levels of OPN and miR-181a had an inverse correlation in the serum of children with AR.

OPN is expressed in a variety of cells, including bronchial epithelial cells, airway and vascular smooth muscle cells, myofibroblasts, and inflammatory cells like T-lymphocytes and mast cells [16]. The elevated levels of OPN were observed in IgE-mediated allergic inflammation, such as in serum, sputum supernatant and bronchoalveolar lavage fluid from asthmatics [16–19], nasal mucosa from allergic rhinitis [20, 21], and tear fluid from allergic conjunctivitis [22]. We confirmed the serum level of OPN was increased in adult patients with AR in our previous study [8]. In the present investigation, we found that OPN was increased in the serum of pediatric AR. This was in accordance with the study in preschool children, which indicated that the serum OPN levels of the asthmatic children with AR were higher than those of the patients without AR [9], confirming the involvement of OPN in pediatric AR.

The biological functions of OPN can be profoundly influenced by posttranslational modifications [23]. Accumulating data have suggested that miRNAs have the potential to regulate OPN expression, such that miR-4262 targeted the 3′-UTR of OPN mRNA to inhibit its translation in osteosarcoma [24], miR-181a in vascular smooth muscles cells inhibited angiotensin II-induced OPN expression in atherosclerosis [12], and miR-181a altered OPN 3′-UTR dependent reporter expression and suppressed OPN expression in hepatocellular cancer cell lines [13]. However, the modification of OPN in the context of allergic airway inflammation by miRNAs remains unclear. Altered miRNAs profiles in nasal mucosa and bronchoalveolar lavage fluid exosomes in allergic airway inflammation have been reported in several studies [11, 25, 26]. Therefore, one might hypothesize that OPN production in serum from AR children was affected by miRNAs. In this study, we found that the serum level of miRNA-181a was decreased. We also found an inverse correlation between serum OPN and miRNA-181a levels among children with AR. However, the ultimate reason for changes in level of systemic OPN in AR children has not yet been identified. Further studies are required to explore whether there is a cause-effect relationship between OPN and miR-181a in the serum from children with AR.

A number of studies have recognized the important roles of OPN in Th1-associated immunological diseases such as Crohn's disease [27] and tuberculosis [28] and Th17 cells participated diseases such as rheumatoid arthritis [29]. It has been also implicated in the development of autoimmune diseases (systemic lupus nephritis) that are mediated by Th2 cells [30, 31]. However, contradictory findings exist regarding the roles of OPN in different phase and type of allergic diseases. For example, in allergic asthma, OPN enhances sensitization but downmodulates Th2-driven IL-4-dominated inflammation. In Th1-driven delayed-type allergy, such as allergic contact dermatitis, OPN supports dendritic cell migration and IL-12 expression and is secreted by T effector cells and keratinocytes, augmenting Th1-mediated allergy and supporting disease chronification. A recent study demonstrated that OPN has a proinflammatory role during the primary (sensitization) phase but an anti-inflammatory and protective effect during antigenic challenge [23]. Those above different roles of OPN might in part contribute to the multifunctional nature of OPN [6] or several isoforms of OPN like intracellular (iOPN) and secreted (sOPN) ones [23].

Our present study in pediatric AR demonstrated a positive correlation between OPN and IL-4, a representative cytokine of Th2-mediated allergic responses, and negative correlation with Th1 cytokine IFN-*γ*. The findings are supported by previous observations demonstrating that serum OPN concentration was correlated with blood serum ECP and IL-5 levels which were also typical Th2 cytokines in adult AR [8]. Furthermore, in a study using airway epithelial cell line BEAS-2B cells, OPN has been related to the unregulated mRNA expression of IL-4, IL-5, and IL-13 [21]. Meanwhile, serum miR-181a level was negatively correlated with IL-4 and positively correlated with IFN-*γ*. Based on the above analysis, it seems plausible that miRNA-181a acted on OPN to modulate the expression of pro- and anti-inflammatory cytokines in the setting of childhood AR.

We emphasize that the altered systemic OPN and miR-181a are related to the modulation of Th2-mediated allergic immune response. Therefore, it is reasonable to ask whether both of them contribute to the severity of AR in clinical scenario. In the present study, serum OPN showed a significant positive relationship and miR-181a had an inverse correlation with total nasal symptom scores in pediatric AR patients. These findings suggest that OPN plays a crucial role in driving the progression of childhood AR and miR-181a is more likely to have opposing effect. Nonetheless, the question of whether decreased miR-181a mediated posttranscriptional silencing is a potential mechanism for the upregulation of OPN needs further investigation.

## 5. Conclusions

In summary, children with AR showed a marked increase in serum level of OPN but a decrease in miR-181a level. Our findings may be beneficial for designing a potential strategy for the optimal prevention and management of AR children.

## Figures and Tables

**Table 1:** Demographic characteristics of AR children and normal controls.

Groups	AR group	Control
Number	25	20
Sex (male : female)	13 : 12	11 : 9
Age (months)	65.2 ± 34.0	73.8 ± 21.9
History of asthma, *n* (%)	0	0
ECP^a^ (ng/mL)	38.5 (23.0–124.0)^*∗*^	8.9 (3.6–18.0)
IgEa (IU/mL)	139.1 (29.5–1013.0)^*∗*^	21.3 (3.3–39.0)

^a^Data presented as median values (minimum-maximum).

^*∗*^Compared with control group, *P*<0.05.

ECP: eosinophil cationic protein.

**Table 2:** Th cytokines of AR children and normal controls.

Cytokines	AR group	Control
IL-4 (pg/mL)	6.23 ± 1.13^*∗*^	2.24 ± 0.36
IL-5 (pg/mL)	55.1 ± 21.9^*∗*^	9.23 ± 1.56
IFN-*γ* (pg/mL)	24.8 ± 8.8^*∗*^	56.7 ± 14.6
IL-12 (pg/mL)	3.15 ± 0.35^*∗*^	6.56 ± 2.08

^*∗*^Compared with control group, *P* < 0.05.

**Table 3:** Relationship between OPN/miR-181a and Th1/2 cytokines.

	OPN		miR-181a	
	*r*	*P*	*r*	*P*
IL-4	0.653	0.02	−0.65	0.01
IL-5	0.581	0.01	−0.724	0.03
IFN-*γ*	−0.595	0.03	0.623	0.02
IL-12	−0.713	0.02	0.754	0.04

## Abbreviations

AR: Allergic rhinitis
IL: Interleukin
OPN: Osteopontin
qPCR: Quantitative polymerase chain reaction
TNSS: Total nasal severity score.
